# Supplementary material for: The Inhibiting Effect of GB-2, (+)-Catechin, Theaflavin, and Theaflavin 3-Gallate on Interaction between ACE2 and SARS-CoV-2 EG.5.1 and HV.1 Variants
Source: Int J Mol Sci. 2024 Aug 31;25(17):9498. doi: 10.3390/ijms25179498 (PMC11394907; doi:10.3390/ijms25179498)
Supplement: Supplementary file 1 [file ijms-25-09498-s001.zip › ijms-3126592-supplementary.pdf]

Figure legend

Supplement Figure S1. (A, B)The RetTime, width, area, height, and area of HPLC chromatograms in Fig. 1E and F, respectively.

Supplement Figure S1. A GB-2

Signal 1: DAD1 C, Sig=278,2 Ref=360,100

Peak #	RetTime [min]	Type	Width [min]	Area [mAU*s]	Height [mAU]	Area %
1	2.463	BV	0.0921	457.73547	72.10011	0.8594
2	2.644	VV	0.1313	901.27087	94.50438	1.6922
3	2.836	VV	0.1179	760.09320	86.99858	1.4271
4	3.095	VV	0.1337	245.71352	28.18625	0.4613
5	3.273	VV	0.0774	967.16492	196.60808	1.8159
6	3.515	VV	0.1787	2884.78760	257.45313	5.4163
7	3.828	VV	0.1845	1125.72375	86.09402	2.1136
8	4.456	VV	0.5796	224.14316	4.74057	0.4208
9	5.827	VV	0.5052	404.10913	10.13551	0.7587
10	7.285	VV	0.2929	3.87234e4	1875.35803	72.7050
11	8.236	VB	0.2988	303.82419	13.69685	0.5704
12	10.028	BV	0.3384	175.55540	6.81704	0.3296
13	10.272	VV	0.3507	218.31638	8.47035	0.4099
14	10.940	VP	0.3097	244.84979	10.83105	0.4597
15	12.112	PV	0.2443	31.95530	1.78986	0.0600
16	12.650	VP	0.3181	64.63617	2.87697	0.1214
17	13.628	BB	0.4277	86.41125	2.64086	0.1622
18	15.815	PV	0.3065	200.79611	8.47216	0.3770
19	16.393	VP	0.4341	2222.58521	68.21485	4.1730
20	17.753	BV	0.4069	103.13314	3.26162	0.1936
21	18.450	BB	0.2472	56.06297	3.12725	0.1053
22	20.963	BV	0.3750	310.29245	11.71464	0.5826
23	21.471	VV	0.3007	367.11319	15.94033	0.6893
24	21.990	VV	0.2457	397.98230	20.75890	0.7472
25	22.403	VP	0.1700	170.47560	13.61977	0.3201
26	22.720	VV	0.1435	199.75963	20.17543	0.3751
27	22.832	VV	0.1539	255.71371	21.95630	0.4801
28	23.218	VV	0.2259	511.72760	29.87099	0.9608
29	23.617	VV	0.1983	116.11818	7.95684	0.2180
30	23.843	VB	0.2408	103.25902	5.45645	0.1939
31	24.732	BB	0.1818	16.64766	1.24632	0.0313
32	25.168	BV	0.0941	46.28460	7.29042	0.0869

(+)-catechin

Signal 1: DAD1 C, Sig=278,2 Ref=360,100

Peak #	RetTime [min]	Type	Width [min]	Area [mAU*s]	Height [mAU]	Area %
1	2.736	VV	0.0698	14.12286	2.84867	0.1219
2	2.908	VV	0.1767	75.75338	5.36656	0.6536
3	3.168	VP	0.0967	52.82352	8.03785	0.4558
4	3.281	VV	0.0435	14.11447	4.91550	0.1218
5	7.323	BP	0.4004	1.14329e4	432.45966	98.6470

T3G

Signal 1: DAD1 C, Sig=278,2 Ref=360,100

Peak #	RetTime [min]	Type	Width [min]	Area [mAU*s]	Height [mAU]	Area %
1	3.397	VB	0.2131	205.12631	12.52482	2.4576
2	15.794	BV	0.8387	8108.87646	135.40335	97.1526
3	17.480	VB	0.2819	32.53094	1.51881	0.3898

Theaflavin

Signal 1: DAD1 C, Sig=278,2 Ref=360,100

Peak #	RetTime [min]	Type	Width [min]	Area [mAU*s]	Height [mAU]	Area %
1	3.404	VV	0.1241	91.96664	9.92457	1.7685
2	3.582	VB	0.2840	395.96576	20.99529	7.6143
3	14.577	BP	0.9637	4712.35205	72.37125	90.6172

Supplement Figure S1.

B **GB-2**

Peak #	RetTime [min]	Type	Width [min]	Area [mAU*s]	Height [mAU]	Area %
1	2.748	BV	0.3015	1.94901e4	856.38727	20.0726
2	3.012	VV	0.1092	7976.50732	998.56757	8.2149
3	3.211	VV	0.1619	2.89449e4	2380.12744	29.8101
4	3.501	VV	0.1087	1.84948e4	2326.59082	19.0476
5	3.764	VV	0.1121	988.62115	125.18862	1.0182
6	4.045	VV	0.1970	2005.92883	141.79774	2.0659
7	4.249	VV	0.2528	2597.75269	136.05379	2.6754
8	4.983	VV	0.2080	375.78558	26.09617	0.3870
9	5.262	VV	0.2484	458.25961	27.22337	0.4720
10	5.529	VV	0.1836	304.31723	24.06096	0.3134
11	5.805	VV	0.2089	583.57642	40.31078	0.6010
12	6.072	VV	0.3369	771.42560	32.50219	0.7945
13	6.820	VB	0.4483	259.04431	7.90553	0.2668
14	8.582	PV	0.2601	197.02327	11.15343	0.2029
15	8.964	VV	0.3763	248.16158	9.33078	0.2556
16	9.810	VV	0.2821	43.23309	2.31257	0.0445
17	10.338	VP	0.5257	81.92257	2.32158	0.0844
18	12.368	BV	0.4110	5556.13818	198.25616	5.7222
19	12.870	VB	0.4692	4808.80322	153.63423	4.9525
20	19.431	PV	0.5899	1610.36780	39.98472	1.6585
21	20.166	VB	0.6887	1301.13184	28.82239	1.3400

Glycyrrhizic acid

Peak #	RetTime [min]	Type	Width [min]	Area [mAU*s]	Height [mAU]	Area %
1	2.872	BP	0.1336	66.59571	7.96324	0.5891
2	3.161	VV	0.1223	66.30411	7.41343	0.5865
3	3.397	VB	0.2632	172.08714	9.41811	1.5222
4	12.461	BB	0.8743	1.10000e4	166.16432	97.3022
